# Single cell genomes of *Prochlorococcus*, *Synechococcus*, and sympatric microbes from diverse marine environments

**DOI:** 10.1038/sdata.2018.154

**Published:** 2018-09-04

**Authors:** Paul M. Berube, Steven J. Biller, Thomas Hackl, Shane L. Hogle, Brandon M. Satinsky, Jamie W. Becker, Rogier Braakman, Sara B. Collins, Libusha Kelly, Jessie Berta-Thompson, Allison Coe, Kristin Bergauer, Heather A. Bouman, Thomas J. Browning, Daniele De Corte, Christel Hassler, Yotam Hulata, Jeremy E. Jacquot, Elizabeth W. Maas, Thomas Reinthaler, Eva Sintes, Taichi Yokokawa, Debbie Lindell, Ramunas Stepanauskas, Sallie W. Chisholm

**Affiliations:** 1 Department of Civil and Environmental Engineering, Massachusetts Institute of Technology, Cambridge, MA 02139, USA.; 2 Department of Systems and Computational Biology, Department of Microbiology and Immunology, Albert Einstein College of Medicine, Bronx, NY 10461, USA.; 3 Department of Limnology and Bio-Oceanography, University of Vienna, Vienna 1090, Austria.; 4 Department of Earth Sciences, University of Oxford, Oxford OX1 3AN, UK.; 5 Marine Biogeochemistry Division, GEOMAR Helmholtz Centre for Ocean Research, Kiel 24148, Germany.; 6 Research and Development Center for Marine Biosciences, Japan Agency for Marine-Earth Science and Technology, Yokosuka 237-0061, Japan.; 7 Department F.-A. Forel for Environmental and Aquatic Sciences, University of Geneva, Geneva 1211, Switzerland.; 8 Faculty of Biology, Technion–Israel Institute of Technology, Haifa 3200003, Israel.; 9 Department of Biological Sciences, University of Southern California, Los Angeles, CA 90089, USA.; 10 Ministry for Primary Industries, Napier 4144, New Zealand.; 11 Single Cell Genomics Center, Bigelow Laboratory for Ocean Sciences, East Boothbay, ME 04544, USA.; 12 Department of Biology, Massachusetts Institute of Technology, Cambridge, MA 02139, USA.

**Keywords:** Microbial ecology, Coevolution, Biogeography, Marine biology, Genome

## Abstract

*Prochlorococcus* and *Synechococcus* are the dominant primary producers in marine ecosystems and perform a significant fraction of ocean carbon fixation. These cyanobacteria interact with a diverse microbial community that coexists with them. Comparative genomics of cultivated isolates has helped address questions regarding patterns of evolution and diversity among microbes, but the fraction that can be cultivated is miniscule compared to the diversity in the wild. To further probe the diversity of these groups and extend the utility of reference sequence databases, we report a data set of single cell genomes for 489 *Prochlorococcus*, 50 *Synechococcus*, 9 extracellular virus particles, and 190 additional microorganisms from a diverse range of bacterial, archaeal, and viral groups. Many of these uncultivated single cell genomes are derived from samples obtained on GEOTRACES cruises and at well-studied oceanographic stations, each with extensive suites of physical, chemical, and biological measurements. The genomic data reported here greatly increases the number of available *Prochlorococcus* genomes and will facilitate studies on evolutionary biology, microbial ecology, and biological oceanography.

## Background & Summary

Marine cyanobacteria within the genera *Prochlorococcus* and *Synechococcus* are estimated to be responsible for roughly 25% of ocean net primary productivity^1^. *Prochlorococcus* is the numerically dominant phototroph in oligotrophic subtropical gyres, which are among the largest contiguous biomes on Earth^2^. In these nutrient poor regimes, *Prochlorococcus* can account for over half of the chlorophyll^3,4^. While *Prochlorococcus* is generally restricted to open ocean habitats between 45^o^N and 40^o^S, *Synechococcus* has a much broader geographical distribution that extends to subpolar and coastal regions^1^. This difference in range is thought to be due in part to the greater phenotypic flexibility and regulatory capacity among *Synechococcus*, enabling acclimation to heterogeneous conditions^5^. By contrast, *Prochlorococcus* has a more streamlined genome adapted to less variable but nutrient depleted regions of the open ocean^6^. Although *Prochlorococcus* cells have the smallest genomes of known oxygenic phototrophs (~1.6–2.7 Mbp and ~2000–3000 genes), the global collective of this group harbors an immense diversity of protein encoding genes^7^. Recent estimates using 41 genomes of cultivated isolates suggested that the *Prochlorococcus* pan-genome–the complete set of genes harbored by all *Prochlorococcus*–contains more than 80,000 distinct genes^6^, many of which presumably play a role in adaptation to local environmental conditions. Only a small fraction of these genes have been catalogued, highlighting the potential for culture-independent single cell genomics to reveal new ecologically relevant functions among *Prochlorococcus*.

As a consequence of their abundance and global distribution, *Prochlorococcus* and *Synechococcus* perform key functions at the base of marine food webs, primarily the supply of fixed carbon to higher trophic levels. Much of this carbon is regenerated through respiration by co-occurring heterotrophic bacteria, such as the highly abundant SAR11 clade of marine Alphaproteobacteria (*Candidatus* Pelagibacter ubique). Many of these heterotrophic bacteria perform ecosystem services that in turn benefit the cyanobacterial populations. In particular, *Prochlorococcus* is highly sensitive to reactive oxygen species, such as hydrogen peroxide^8^, which can be detoxified by some heterotrophic community members. Abundant catalase encoding heterotrophs in oligotrophic environments, such as the SAR86 and SAR116 clades of marine proteobacteria^9,10^ and some sub-populations of SAR11 (ref. 11), are likely to be important community members that provide cross-protection for sensitive *Prochlorococcus* populations. Recent work further suggests that *Prochlorococcus* and the dominant heterotrophic cells in the oligotrophic ocean have evolved metabolic co-dependencies to maximize metabolic potential^11^. Thus, understanding the diversity of functions performed by sympatric heterotrophic cells is essential for understanding the ecology and evolution of *Prochlorococcus* and the combined impact these microbial groups have on ecosystem function and ocean biogeochemistry.

Although studies using cultivated isolates have revealed much about the ecology of *Prochlorococcus*, *Synechococcus*, SAR11, and other important taxa that contribute to the function of marine ecosystems^12^, culture-independent studies have begun to reveal an astounding degree of diversity in the wild. In particular, recent advances in the genomics of single cells have uncovered previously unknown marine microbial phyla and functions^13^ and have identified a high degree of genome streamlining, mixotrophy, and metabolic specialization within bacterial cells of the surface ocean^14^. Single cell genomes of *Prochlorococcus* have revealed the existence of new clades with distinct ecological and physiological adaptations^15^ as well as a high degree of genomic and functional diversity among *Prochlorococcus* cells with nearly identical ribotypes^16,17^.

**Figure 1:**
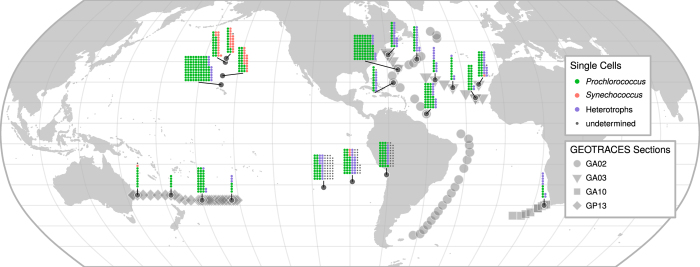
Map of sampling locations. Single cell genomes at each site are represented by miniaturized stacked dot-plots (each dot represents one single cell genome), with organism group indicated by color, and cells categorized as “undetermined” if robust placement within known phylogenetic groups failed due to low assembly completeness/quality or missing close references. Larger points correspond to stations on associated GEOTRACES cruises.

Single cell genomes of both abundant and rare taxa are useful for phylogenetic anchoring of metagenomic data sets and expanding our knowledge of previously undetected phylogenetic lineages and the functions they harbor. Casting a broad net in order to most effectively capture the diversity of cyanobacterial and sympatric heterotrophic microorganisms, we have obtained samples from 22 geographic locations across the world’s oceans (Fig. 1), representing 10 Longhurst biogeographical provinces^18,19^ (Table 1). Many of these samples were collected under the auspices of the BioGEOTRACES component of GEOTRACES^20^. From these samples, we report 738 single cell genome assemblies consisting of 489 *Prochlorococcus*, 50 *Synechococcus*, 82 SAR11, 17 SAR116, 16 SAR86, 9 extracellular virus particles, and 75 additional sympatric microorganisms. To aid in the identification of orthologous genes and facilitate comparative genomics studies, we have precomputed a set of cyanobacterial and cyanophage specific clusters of orthologous groups of proteins (CyCOGs). We expect these data to be useful for a variety of studies related to evolutionary biology, microbial ecology, and ocean biogeochemistry.

## Methods

### Sample collection

Samples were collected on 13 cruises in the Pacific and Atlantic oceans and encompass 30 discrete biosamples (Table 1) from the following Longhurst Provinces: CHIL, Coastal-Chile-Peru Current Coastal Province; SPSG, Westerlies-S. Pacific Subtropical Gyre Province; NPTG, Trades-N. Pacific Tropical Gyre Province; NASW, Westerlies-N. Atlantic Subtropical Gyral Province (West) (STGW); NATR, Trades-N. Atlantic Tropical Gyral Province (TRPG); GFST, Westerlies-Gulf Stream Province; EAFR, Coastal-E. Africa Coastal Province; AUSE, Coastal-East Australian Coastal Province; ARCH, Trades-Archipelagic Deep Basins Province; NPPF, Westerlies-N. Pacific Polar Front Province. A minimum of 2 replicates of 1-2 mL of raw seawater was transferred to sterile cryovials with glycerol added as a cryoprotectant at a final concentration of 10%. Samples were flash frozen in liquid nitrogen and stored at -80 °C.

### Single amplified genome (SAG) generation

The generation, identification, sequencing, and *de novo* assembly of SAGs were performed at the Bigelow Laboratory for Ocean Sciences’ Single Cell Genomics Center (scgc.bigelow.org). The cryopreserved samples were thawed and pre-screened through a 40 μm mesh size cell strainer (Becton Dickinson). Fluorescence-activated cell sorting (FACS) was performed using a BD InFlux Mariner flow cytometer equipped with a 488 nm laser for excitation and a 70 μm nozzle orifice (Becton Dickinson, formerly Cytopeia), as previously described^16,21^. The cytometer was triggered on side scatter, and the “single-1 drop” mode was used for maximal sort purity. For cyanobacteria, the sort gate was defined based on cellular pigment autofluorescence^16^. In order to discriminate heterotrophic bacteria and extracellular particles, environmental samples were incubated with the SYTO-9 DNA stain (5 μM final concentration; Thermo Fisher Scientific) for 10–60 min, after which the particle green fluorescence (proxy to nucleic acid content), light side scatter (proxy to size), and the ratio of green versus red fluorescence (for improved discrimination of cells from detrital particles) were used to define the sort gate^21^. Individual cells were deposited into 384-well plates (Table 2) containing 600 nL per well of 1x TE buffer and stored at -80°C until further processing. Of the 384 wells, 317 wells were dedicated for single particles, 64 wells were used as negative controls (no droplet deposition), and 3 wells received 10 particles each to serve as positive controls. BD FACS Sortware software was used to collect index sort data (indexed_facs_wga_summary.tsv, Data Citation 1), with FACS plots available from figshare (facs_ssc_fsc_plots.pdf, Data Citation 1). Diameters of sorted cells (indexed_facs_wga_summary.tsv, Data Citation 1) were determined using the FACS light forward scatter signal, which was calibrated against cells of microscopy-characterized laboratory cultures^21^. The DNA for each cell was amplified using either multiple displacement amplification (MDA) or WGA-X^21^, with amplification kinetics distributions for each plate available from figshare (kinetics_welltype_distributions_summary.pdf, Data Citation 1).

### Marker gene screening

Single cell MDA and WGA-X products were diluted 50x in UV-treated, 0.2 μm filtered water and then used as templates in real-time PCR, as previously described^21^. Heterotrophic bacteria were screened using 16S rRNA gene primers 27 F and 907 R. Cyanobacteria were analyzed using primers targeting the 16S-23S intergenic transcribed spacer (ITS) sequence^16^. The obtained PCR amplicons were sequenced from both ends using Sanger technology at GeneWiz (South Plainfield, NJ). The two reads were automatically aligned and the consensus was manually curated using Sequencher v4.7 (Gene Codes Corporation, Ann Arbor, MI, USA). Chimeric 16S rRNA sequences were identified using DECIPHER^22^ and removed. ITS and 16S rRNA sequences have been deposited with GenBank (Data Citation 2,Data Citations 3).

### Cell selection

A selection of cyanobacterial and extracellular SAGs derived from the BiG-RAPA cruise (plates AG-311, AG-315, AG-321, AG-331, AG-335, AG-341, AG-316, AG-323, AG-339, and AG-345) and HOT and BATS cruises (plates AG-347, AG-355, AG-363, and AG-402) were chosen for sequencing based on their fast whole genome amplification, which correlates with good genome recovery in de novo assemblies^21^. Forty-eight additional cyanobacterial SAGs from plates AG-347, AG-355, AG-363, AG-402, AG-418, and AG-459 were selected based on the presence/absence of the *narB* marker gene as determined by a PCR screen using primer sequences 5’-CANTGGCAYACNATGAC-3’ and 5’-RAANCCCCARTGCATNGG-3’. All other cyanobacterial SAGs were selected based on ITS taxonomy with the aim of obtaining a diverse set of cyanobacterial single cell genomes from multiple geographic locations and depths. All heterotroph SAGs were selected based on the classification of 16S sequences using the Ribosomal Database Project (RDP) Release 11 (ref. 23). We focused on obtaining a diverse pool of heterotrophs, including those with poor representation in public databases, that co-occur with *Prochlorococcus* and *Synechococcus* (e.g. SAR11, SAR116, SAR86, marine Actinobacteria, OCS116, SAR202, and SAR324).

### Genomic sequencing and *de novo* assembly

Illumina libraries were created, sequenced and de novo assembled as previously described^21^. Only contigs longer than 2,000 bp were retained. This workflow was evaluated for assembly errors using three bacterial benchmark cultures with diverse genome complexity and %GC, indicating 60% average genome recovery, no non-target and undefined bases, and average frequencies of misassemblies, indels and mismatches per 100 kbp: 1.5, 3.0 and 5.0 (ref. 21). Paired-end sequencing reads (Data Citation 4) and genome assemblies (Data Citations 5–7) have been deposited with NCBI.

### Genome annotation

All genome assemblies were also deposited at the Joint Genome Institute’s Integrated Microbial Genomes (IMG) system and annotated using the JGI Microbial Genome Annotation Pipeline (MGAP v. 4)^24,25^. Assembled genome sequences, gene calls, and functional annotations are available from figshare (Data Citation 1) and IMG (https://img.jgi.doe.gov/). Data can also be viewed and analyzed within IMG/ProPortal (https://img.jgi.doe.gov/proportal). A table linking IMG accession numbers with genome assembly statistics is provided to facilitate use of these annotation data (genome_assembly_summary.tsv, Data Citation 1).

### Phylogeny

**Figure 2:**
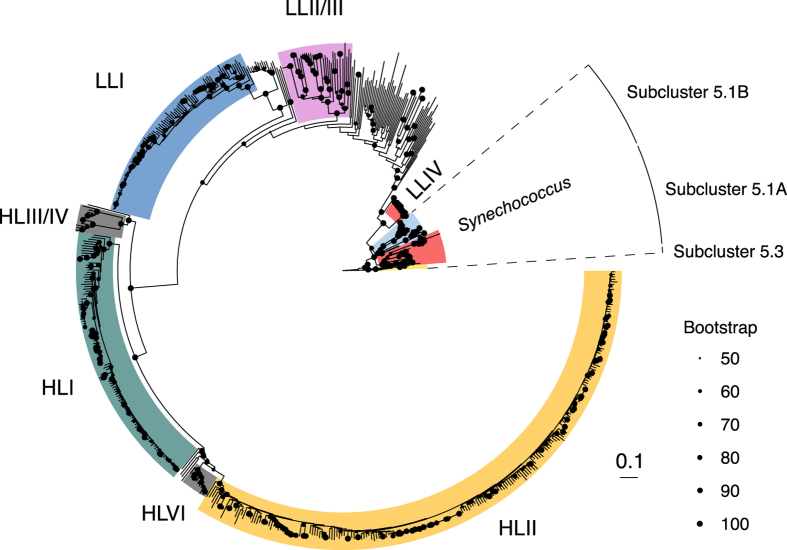
Maximum Likelihood phylogeny of cyanobacterial genomes. The phylogeny includes 66 *Prochlorococcus* isolate genomes, 27 *Synechococcus* isolate references, and 588 single cell genomes (533 of which are part of this project). Bootstrap values are represented by size-scaled dots at nodes. Bootstrap values less than 50 are omitted. Scale bar represents 0.1 nucleotide substitutions per sequence position. Phylogenetic clade membership is indicated by colored blocks and text labels. The three *Synechococcus* subclusters displayed are highlighted by dashed lines and a segmented outer ring. The tree is rooted at *Synechococcus* sp. WH5701 (subcluster 5.2). The underlying data set used for phylogenetic inference was a concatenated alignment of 2–37 PhyloSift marker gene families (see methods for details).

**Figure 3:**
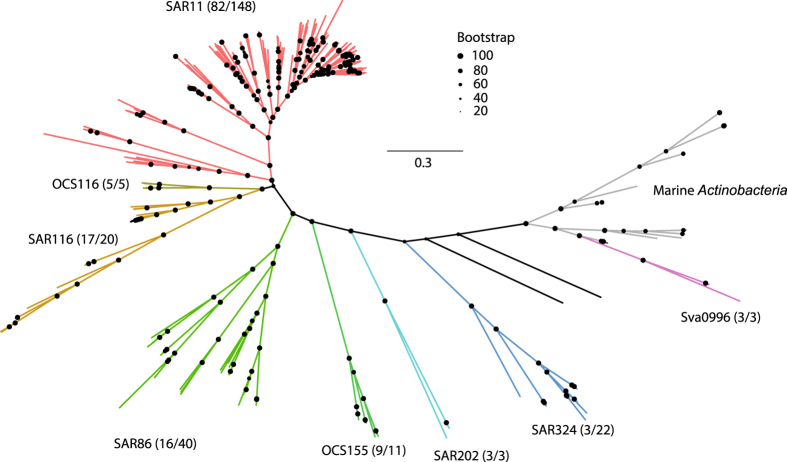
Maximum Likelihood phylogeny of heterotrophic bacterial single cell genomes and additional reference genomes. Bootstrap values are represented by size-scaled dots at nodes. Scale bar represents 0.3 nucleotide substitutions per sequence position. The eight taxonomic lineages of the single cells are colored and labeled. Additional marine *Actinobacteria* lineages are presented in grey to provide added context for the Sva0996 lineage. Numbers in parenthesis indicate the number of single cell genomes from each lineage relative to the total number of genomes in that lineage used to construct the tree. The underlying data set used for phylogenetic inference was a concatenated alignment of 2-37 PhyloSift marker gene families (see methods for details).

In order to facilitate downstream analyses, we have inferred the phylogeny for the cyanobacterial genomes and heterotrophic bacterial genomes (Figs. 2 and 3). We used the PhyloSift software^26^ to identify and align a collection of core protein coding gene families from the single cell genomes. Briefly, PhyloSift uses LAST^27^ to identify 37 protein-coding marker genes^28^. The identified orthologous sequences are then aligned to marker gene HMM profiles using the hmmer software suite^29^ and concatenated into a reading-frame-aware nucleotide codon alignment. The alignments were then trimmed using the automated heuristic method -automated1 in trimAl v1.2 (ref. 30). The recovery of PhyloSift marker gene families ranged from 2–37 (median 31) families per heterotroph single cell genome and from 0–37 (median 27) families per cyanobacterial single cell genome. For phylogenetic inference, we included all single cell genomes with at least 2 PhyloSift marker families present in the final alignments, and 151 additional heterotroph genomes to provide greater context for the heterotroph tree (Data Citation 1). We made our marker-family selection criteria as inclusive as possible in order to convey general tree topology for the greatest possible proportion of the dataset, but we note that some of the most incomplete genomes may be subject to phylogenetic artefacts due to the large number of gaps in their alignments. Maximum Likelihood trees were inferred using raxmlHPC-PTHREADS-AVX v8.2.9 (ref. 31) using the GTRGAMMA model of rate heterogeneity for heterotrophs and the GTRCAT model for cyanobacteria. RAxML runs were conducted with rapid bootstrapping^32^, and the number of bootstrap trees was automatically determined using the extended majority rule criterion^33^ resulting in 300 and 100 bootstrap replicates for the cyanobacterial and heterotroph trees respectively. Lists of taxa used for the phylogenies as well as the alignments in FASTA format and trees in Newick format are available from figshare (Data Citation 1).

### Cyanobacterial Clusters of Orthologous Groups of proteins (CyCOGs)

To provide an overview of functional composition of the genes present in the cyanobacterial and cyanophage genomes, we inferred clusters of orthologous groups of proteins, referred to here as CyCOGs. The underlying set of genomes includes *Prochlorococcus*, *Synechococcus,* cyanophages, and cyanobacterial virocells (genome assemblies containing both bacteria and phage genomes) from our data set. We also included publicly available genome data from IMG for *Prochlorococcus*, marine *Synechococcus* in subclusters 5.1, 5.2, and 5.3, and cyanophages isolated using these cyanobacteria as hosts. The following assemblies with likely heterotroph contamination were excluded: AG-418-M21 and scB245a_518D8 (ref. 16).

The clustering of the proteins was carried out with panX^34^, with parameters tuned to account for incompleteness of SAG genomes: --core_genome_threshold 0.5 --core_gene_strain_fpath strains_complete_95plus.txt (core genes are defined by being present in at least 50% of all genomes, and in every genome of >95% completeness). The workflow comprises an initial clustering step performed with MCL^35^ on all-versus-all alignments generated with DIAMOND^36^, followed by a phylogeny-aware postprocessing procedure to split paralogous groups^34^. This analysis yielded a total of 40,295 CyCOGs (cycogs.tsv, Data Citation 1), of which 23,427 are found in *Prochlorococcus*, 17,692 are found in *Synechococcus*, and 3,267 are found in cyanophage.

This is ProPortal CyCOGs version 6.0 (cycogs.tsv, Data Citation 1). Prior releases include versions 1, 3, 4, and 5 of ProPortal CyCOGs^37–40^. Version 2 is an unreleased set of CyCOGs developed for testing purposes only. Legacy CyCOG definitions are also available from IMG/ProPortal (https://img.jgi.doe.gov/proportal).

### Code availability

No custom code was used in the generation or processing of the data. Software versions and the use of any adjustable variables and parameters are as follows:

DECIPHER 2.2.0 (ref. 22)

Trimmomatic 0.32 (ref. 41): -phred33 LEADING:0 TRAILING:5 SLIDINGWINDOW:4:15 MINLEN:36

kmernorm 1.05: -k 21 -t 30 -c 3 (http://sourceforge.net/projects/kmernorm)

SPAdes 3.0.0 (ref. 42): --careful --sc --phred-offset 33

SPAdes 3.9.0 (ref. 42): --careful --sc --phred-offset 33

PhyloSift 1.0.1 (ref. 26): search --isolate --besthit --threads 20

PhyloSift 1.0.1 (ref. 26): align --isolate --besthit --threads 20

trimAl 1.2 (ref. 30): -automated1

RAxML 8.2.9 (ref. 31): -T 20 -m GTRCAT -p 8048 -f a -x 39381 -# autoMRE

RAxML 8.2.9 (ref. 31): -T 20 -m GTRGAMMA -p 82748 -f a -x 34671 -# autoMRE

## Data Records

File 1: Fluorescence-activated cell sorting (FACS) plots associated with the single cell genome assemblies can be found in facs_ssc_fsc_plots.pdf (Data Citation 1).

File 2: DNA amplification kinetics summaries associated with the single cell genome assemblies can be found in kinetics_platemap_summary.pdf (Data Citation 1).

File 3: DNA amplification kinetics distributions associated with the single cell genome assemblies can be found in kinetics_welltype_distributions_summary.pdf (Data Citation 1).

File 4: A complete list of genomes used for CyCOG annotations can be found in cycogs-genomes.tsv (Data Citation 1).

IID – Strain or single cell identifier

GROUP – *Prochlorococcus*, *Synechococcus*, or Virus

IMG_ID – IMG genome identification number

TYPE – Single amplified genome (SAG) or cultured reference (ISOLATE)

JGI_GENOMEPORTAL_NAME – Name in the JGI Genome Portal

Completeness – Percent genome completeness determined by checkM

File 5: CyCOG definitions can be found in cycogs.tsv (Data Citation 1).

cycog_iid – Unique CyCOG identifier

cycog_num_taxa – Number of genomes containing CyCOG

cycog_num_genes – Number of genes encompassed by CyCOG

cycog_num_duplications – Number of paralogous genes within CyCOG

cycog_num_pro – Number of *Prochlorococcus* genes within CyCOG

cycog_num_syn – Number of *Synechococcus* genes within CyCOG

cycog_num_phage – Number of cyanophage/virus genes within CyCOG

cycog_cns_product – Consensus annotation for genes within CyCOG

cycog_genes – Comma delimited list of all genes found in CyCOG with the format of A_B, where A is the IID of the genome found in File 4 and B is the unique IMG gene ID.

File 6: Taxa used for the phylogeny of cyanobacteria can be found in cyanobacteria_phylogeny_taxa.tsv (Data Citation 1).

File 7: The reading-frame-aware nucleotide codon alignment used for phylogenetic inference of cyanobacterial taxa can be found in cyanobacteria_phylogeny_alignment.fna (Data Citation 1).

File 8: The maximum likelihood phylogenetic tree for cyanobacteria can be found in cyanobacteria_phylogeny_rootedtree.nwk (Data Citation 1).

File 9: Taxa used for the phylogeny of heterotrophic bacteria can be found in heterotroph_phylogeny_taxa.tsv (Data Citation 1).

File 10: The reading-frame aware nucleotide codon alignment used for phylogenetic inference of heterotrophic bacterial taxa can be found in heterotroph_phylogeny_alignment.fna (Data Citation 1).

File 11: The maximum likelihood phylogenetic tree for heterotrophic bacteria can be found in heterotroph_phylogeny_unrootedtree.nwk (Data Citation 1).

File 12: A bzip2 compressed tar archive containing IMG annotated genome assemblies, gene and protein sequences, and associated annotation files derived from the JGI Microbial Genome Annotation Pipeline (MGAP v. 4)^24,25^ (Data Citation 1).

.fna – Nucleic acid file in multi-fasta format

.genes.fna – Gene sequences in multi-fasta format

.genes.faa – Amino Acids file in mult-fasta format

.gff – Gene Information file in GFF3 format

.cog.tab.txt – COG hits in tab-delimited format

.intergenic.fna – Intergenic regions in multi-fasta format

.ipr.tab.txt – IPR hits in tab-delimited format

.ko.tab.txt – KO and EC annotation in tab-delimited format

.pfam.tab.txt – pFam hits in tab-delimited format

.signalp.tab.txt – Signal peptide annotation in tab-delimited format

.tigrfam.tab.txt – TigrFam hits in tab-delimited format

.tmhmm.tab.txt – Transmembrane helices in tab-delimited format

File 13: Indexed fluorescence activated cell sorting data, estimated cell sizes, and cross-over point (Cp) values for whole genome amplification can be found in indexed_facs_wga_summary.tsv (Data Citation 1).

File 14: IMG genome IDs, phylogenetic inference, usage notes, and genome assembly statistics can be found in genome_assembly_summary.tsv (Data Citation 1).

ITS and 16S sequences for all SAGs (including those that did not undergo whole genome sequencing) are available from GenBank under the accession numbers MG666579-MG668595 for ITS sequences (Data Citation 2) and MH074888-MH077527 for 16S sequences (Data Citation 3).

Paired-end sequencing reads in fastq format are available from the NCBI Sequence Read Archive (Data Citation 4).

Genome assemblies are available from GenBank (Data Citations 5–7).

Annotated genome assemblies and geolocation metadata are available from IMG (https://img.jgi.doe.gov/) and IMG/ProPortal (https://img.jgi.doe.gov/proportal).

## Technical Validation

The quality of the sequencing reads was assessed using fastqc and the quality of the assembled genomes was assessed using checkM^43^ and tetramer frequency analysis as previously described^14^. This workflow was evaluated for assembly errors using a series of benchmark cultures with diverse genome complexity and %GC^21^.

## Usage Notes

While the single cell genomes in this data set were screened for contamination that could have been introduced during cell sorting and DNA amplification, users should be aware that these screening procedures do not eliminate the potential for multiple genomes being present in the same assembly. Some single cell genomes may be derived from cells infected with a bacteriophage (i.e. virocells^44^) and thus contain both host and virus genomes. Other single cells may contain multiple genomes due to a close physical association between two cells that resulted in co-sorting and co-amplification of DNA. Given that many of these events are biologically meaningful, these genome assemblies were not removed from the data set or modified to separate multiple genomes. Based on our technical validation, we have identified possible virocells or co-sorted genomes in the data set (genome_assembly_summary.tsv, Data Citation 1).

Ancillary physical, chemical, and biological data associated with the data set can be accessed from C-MORE (http://hahana.soest.hawaii.edu/cmoreDS/), HOT (http://hahana.soest.hawaii.edu/hot/hot-dogs/), BATS (http://bats.bios.edu/), and GEOTRACES (https://www.bodc.ac.uk/geotraces/data/) using the sample metadata available in Tables 1 and 2. The U.S. Biological and Chemical Oceanography Data Management Office (BCO-DMO) can also be used to access associated data for HOT (https://www.bco-dmo.org/project/2101), BATS (https://www.bco-dmo.org/project/2124), and the U.S. GEOTRACES North Atlantic Transect (https://www.bco-dmo.org/project/2066).

## Additional information

**How to cite this article**: Berube, P. M. *et al*. Single cell genomes of *Prochlorococcus*, *Synechococcus*, and sympatric microbes from diverse marine environments. *Sci. Data* 5:180154 doi: 10.1038/sdata.2018.154 (2018).

**Publisher’s note**: Springer Nature remains neutral with regard to jurisdictional claims in published maps and institutional affiliations.

## Supplementary Material



## Figures and Tables

**Table 1:** Biosamples with associated cruise and geolocation metadata.

**biosample**	**project**	**cruise_name**	**cruise_id**	**station**	**bottle_id**	**depth_m**	**latitude_degrees_north**	**longitude_degrees_east**	**sample_date**	**longhurst_province**
SWC-01	CMORE	BIGRAPA	MV1015	1	100100213	20	−20.08	−70.8	11/19/2010	CHIL
SWC-02	CMORE	BIGRAPA	MV1015	1	100100203	55	−20.08	−70.8	11/19/2010	CHIL
SWC-03	CMORE	BIGRAPA	MV1015	4	100402922	14	−23.46	−88.77	11/29/2010	SPSG
SWC-04	CMORE	BIGRAPA	MV1015	4	100402914	112	−23.46	−88.77	11/29/2010	SPSG
SWC-05	CMORE	BIGRAPA	MV1015	7	100705722	14	−26.25	−103.96	12/8/2010	SPSG
SWC-06	CMORE	BIGRAPA	MV1015	7	100705705	180	−26.25	−103.96	12/8/2010	SPSG
SWC-07	HOT	HOT214	KM0920	ALOHA	2140201221	5	23.75	−158	8/19/2009	NPTG
SWC-08	HOT	HOT216	KOK0917	ALOHA	2160201214	100	23.75	−158	11/4/2009	NPTG
SWC-09	BATS	BATS248	AE0916	BATS	1024800403	10	31.07	−64.17	7/14/2009	NASW
SWC-10	BATS	BATS252	AE0926	BATS	1025200410	100	31.07	−64.17	11/7/2009	NASW
SWC-11	GEOTRACES	GA02(L1)	PE319	16	632891	8	36.2	−53.31	5/20/2010	NASW
SWC-12	GEOTRACES	GA02(L2)	PE321	25	633233	119	24.71	−67.07	6/17/2010	NATR
SWC-13	GEOTRACES	GA02(L2)	PE321	35	634604	100	9.55	−50.47	6/28/2010	NATR
SWC-14	GEOTRACES	GA03(L1)	KN199	7	841892	57.7	24	−22	10/24/2010	NATR
SWC-15	GEOTRACES	GA03(L2)	KN204	4	844349	90.8	38.32	−68.87	11/12/2011	GFST
SWC-16	GEOTRACES	GA03(L2)	KN204	16	845948	89.9	26.14	−44.83	11/30/2011	NASW
SWC-17	GEOTRACES	GA03(L2)	KN204	20	846242	99.7	22.33	−35.87	12/4/2011	NATR
SWC-18	GEOTRACES	GA03(L2)	KN204	24	846716	71.6	17.4	−24.5	12/10/2011	NATR
SWC-19	GEOTRACES	GA10(L1)	D357	9	237839	21.2	−34.98	16.02	11/10/2010	EAFR
SWC-20	GEOTRACES	GP13(L1)	SS2011	4	1223543	50.6	−30	156	5/16/2011	AUSE
SWC-21	GEOTRACES	GP13(L1)	SS2011	22	1222400	50.4	−30	174	5/24/2011	ARCH
SWC-22	GEOTRACES	GP13(L1)	SS2011	38	1224245	76	−32.5	−170	5/31/2011	SPSG
SWC-23	GEOTRACES	GP13(L2)	TAN1109	GT3	1153793	203	−32.5	−170	6/11/2011	SPSG
SWC-24	GEOTRACES	GP13(L2)	TAN1109	GT19	1156283	100	−32.5	−154	6/20/2011	SPSG
SWC-26	SCOPE	GRADIENTS(1.0)	KOK1606	4	10400223	5	28.14	−158	4/22/2016	NPTG
SWC-27	SCOPE	GRADIENTS(1.0)	KOK1606	4	10400206	90	28.14	−158	4/22/2016	NPTG
SWC-28	SCOPE	GRADIENTS(1.0)	KOK1606	6	10600223	5	32.7	−158	4/24/2016	NPPF
SWC-29	SCOPE	GRADIENTS(1.0)	KOK1606	6	10600207	60	32.7	−158	4/24/2016	NPPF
SWC-30	SCOPE	GRADIENTS(1.0)	KOK1606	9	10900223	5	36.57	−158	4/27/2016	NPPF
SWC-31	SCOPE	GRADIENTS(1.0)	KOK1606	9	10900207	65	36.57	−158	4/27/2016	NPPF

**Table 2:** Biosamples and sort gates associated with the plate identification numbers used as prefixes for genome names.

**biosample**	***Prochlorococcus*** **Sort Gate**	***Synechococcus*** **Sort Gate**	**Cyanobacteria Sort Gate**	**Bacteria Sort Gate**	**Extracellular Sort Gate**
SWC-01	AG-311	n/a	n/a	AG-313	n/a
SWC-02	AG-315	AG-316	n/a	AG-319	n/a
SWC-03	AG-321	AG-323	n/a	AG-325	n/a
SWC-04	AG-331	n/a	n/a	AG-333	n/a
SWC-05	AG-335	n/a	n/a	AG-337	AG-339
SWC-06	AG-341	n/a	n/a	AG-343	AG-345
SWC-07	AG-347	n/a	n/a	AG-349	n/a
SWC-08	AG-402	n/a	n/a	AG-404	n/a
SWC-09	AG-355	n/a	n/a	AG-359	n/a
SWC-10	AG-363	n/a	n/a	AG-365	n/a
SWC-11	AG-388	n/a	n/a	AG-390	n/a
SWC-12	AG-412	n/a	n/a	AG-414	n/a
SWC-13	AG-409	n/a	n/a	AG-410	n/a
SWC-14	AG-418	AG-420	n/a	AG-422	n/a
SWC-15	AG-424	n/a	n/a	AG-426	n/a
SWC-16	AG-429	n/a	n/a	AG-430	n/a
SWC-17	AG-432	n/a	n/a	AG-435	n/a
SWC-18	AG-436	n/a	n/a	AG-439	n/a
SWC-19	AG-442	AG-444^a^	n/a	AG-447	n/a
SWC-20	AG-449	AG-450	n/a	AG-453^a^	n/a
SWC-21	AG-455	n/a	n/a	AG-457^a^	n/a
SWC-22	AG-459	n/a	n/a	AG-461	n/a
SWC-23	AG-463	n/a	n/a	AG-464	n/a
SWC-24	AG-469	n/a	n/a	AG-470	n/a
SWC-26	n/a	n/a	AG-670	n/a	n/a
SWC-27	n/a	n/a	AG-673	n/a	n/a
SWC-28	n/a	n/a	AG-676	n/a	n/a
SWC-29	n/a	n/a	AG-679	n/a	n/a
SWC-30	n/a	n/a	AG-683	n/a	n/a
SWC-31	n/a	n/a	AG-686	n/a	n/a

^a^Ribosomal RNA sequences only (16S-23S intergenic transcribed spacer or 16S).
